# Detection of disease in *Cucurbita maxima* Duch. ex Lam*.* caused by a mixed infection of *Zucchini yellow mosaic virus*, *Watermelon mosaic virus*, and *Cucumber mosaic virus* in Southeast China using a novel small RNA sequencing method

**DOI:** 10.7717/peerj.7930

**Published:** 2019-10-23

**Authors:** Yi Wang, Pu Zhu, Qin Zhou, Xiaojun Zhou, Ziqing Guo, Linrun Cheng, Liyan Zhu, Xiaochan He, Yidan Zhu, Yang Hu

**Affiliations:** 1 Jinhua Academy of Agricultural Sciences, Jinhua, Zhejiang, China; 2 Zhejiang Provincial Key Laboratory of Biological and Chemical Utilization of Forest Resouces, Zhejiang Academy of Forestry, Hangzhou, Zhejiang, China

**Keywords:** co-infection, Small RNA sequencing, *Zucchini yellow mosaic virus*, *Watermelon mosaic virus*, And *cucumber mosaic virus*, *Cucurbita maxima*, Diagnostics of virus disease

## Abstract

The genus *Cucurbita* comprises many popular vegetable and ornamental plants, including pumpkins, squashes, and gourds, that are highly valued in China as well as in many other countries. During a survey conducted in Zhejiang province, Southeast China in 2016, severe symptoms of viral infection were observed on *Cucurbita maxima* Duch. ex Lam*.* Diseased plants showed symptoms such as stunting, mosaicking, Shoe string, blistering, yellowing, leaf deformation, and fruit distortion. Approximately, 50% of *Cucurbita* crops produced in Jinhua were diseased, causing an estimated yield loss of 35%. In this study, we developed a method using all known virus genomes from the NCBI database as a reference to map small RNAs to develop a diagnostic tool that could be used to diagnose virus diseases of *C. maxima*. 25 leaf samples from different symptomatic plants and 25 leaf samples from non-symptomatic plants were collected from the experimental field of Jihua National Agricultural Technology Garden for pathogen identification. Small RNAs from each set of three symptomatic and non-symptomatic samples were extracted and sequenced by Illumina sequencing. Twenty-four different viruses were detected in total. However, the majority of the small RNAs were from *Zucchini yellow mosaic virus* (ZYMV),* Watermelon mosaic virus* (WMV), and* Cucumber mosaic virus* (CMV). Mixed infections of these three viruses were diagnosed in leaf samples from diseased plants and confirmed by reverse transcription PCR (RT-PCR) using primers specific to these three viruses. Crude sap extract from symptomatic leaf samples was mechanically inoculated back into healthy *C. maxima* plants growing under greenhouse conditions. Inoculated plants developed the same disease symptoms as those observed in the diseased plants and a mixed infection of ZYMV, WMV, and CMV was detected again by RT-PCR, thus fulfilling Koch’s postulates. The diagnostic method developed in this study involves fewer bioinformatics processes than other diagnostic methods, does not require complex settings for bioinformatics parameters, provides a high level of sensitivity to rapidly diagnose plant samples with symptoms of virus diseases and can be performed cheaply. This method therefore has the potential to be widely applied as a diagnostic tool for viruses that have genome information in the NCBI database.

## Introduction

The Cucurbitaceae family (cucurbits) is a large, genetically diverse group of plants that are highly valued as popular food crops, such as watermelon, zucchini, cucumber, and squashes, and as ornamental plants, such as gourds, in China and in many other countries (Sanjur et al., 2002). Cucurbits are grown in temperate and tropical regions worldwide, where those with edible fruits contribute to the food sector, nutrition, dietary and culinary diversification, health and income generation. However, cucurbits are susceptible to many plant viruses, the majority of which are vector-borne and transmitted by either aphids or whiteflies (Lecoq & Desbiez, 2012).

At least 28 species of virus have been reported to naturally infect cucurbit crops, mainly those that belong to the genera *Crinivirus*, *Begomovirus*, *Ipomovirus*, and *Potyvirus*. Some of these virus species are widely distributed and can cause severe crop losses (Lecoq & Desbiez, 2012; Abrahamian & Abou-Jawdah, 2014; Perotto et al., 2018). New viruses are regularly isolated and almost one ‘new’ cucurbit virus has been reported every 2 years for the past 35 years, although some of these had already been reported on other hosts. Furthermore, the occurrence of single, co-, and triple infections of cucurbit vegetables with *Cucumber mosaic virus* (CMV; a *Cucumovirus*), *Watermelon mosaic virus* (WMV; a *Potyvirus*), and *Zucchini yellow mosaic virus* (ZYMV; a *Potyvirus*) has been reported recently in Karanganyar, Central Java, Indonesia and coastal areas of Tanzania (Sydanmetsa & Mbanzibwa, 2016; Supyani et al., 2017).

Potyviruses among the most important viral pathogens of cucurbits are single-stranded positive-sense RNA viruses that usually have a genome of ∼10 kb and one kind of coat protein. In terms of structure, potyviruses consist of a single flexuous rod-shaped particle 680–900 nanometers long and 12 nanometers in diameter. Like other RNA viruses, the RNA genome of a potyvirus is joined at its 5′ end to a small protein, and its 3′ end has a polyadenylate sequence with approximately 190 adenylates. The main body of a potyvirus can be translated into a huge protein, which is subsequently cleaved at specific points to release eight proteins (Agrios, 2005). The following *Potyvirus* species have been detected worldwide as causal agents of disease in fields of cucurbit crops: *Papaya ringspot virus type Watermelon* (PRSV-W), WMV, and ZYMV. Symptoms of disease in infected plants include mosaicking, mottling, blistering, and leaf and fruit deformations (Lecoq & Desbiez, 2012; Romay, Lecoq, & Desbiez, 2014; Ibaba, Laing, & Gubba, 2016).

In order to defend themselves against virus infection, hosts that have an RNA interference (RNAi) system produce small RNAs of approximately 15–28 bp to further digest the RNA from the virus. RNAi originally referred to the phenomenon in which exogenously introduced double-stranded RNA molecules silenced the expression of homologous gene sequences in the nematode *Caenorhabditis elegans* (Fire et al., 1998). However, recently it has become clear that RNAi is mechanistically related to a number of conserved RNA silencing pathways that are mediated by small noncoding RNAs (Carthew & Sontheimer, 2009; Ghildiyal & Zamore, 2009; Moazed, 2009). Although RNAi pathways comprise different proteins and mechanisms in different organisms, they have some common features (Siomi & Siomi, 2009). The main pathway of RNAi relies on several core proteins such as Dicer and Argonaute. Dicers generate small RNA duplexes from dsRNA precursors, then the dsRNA duplexes are loaded onto the RNA-induced silencing complex (RISC). After the removal of the passenger strand of the dsRNA duplex, the RISC is activated and uses the remaining single-stranded small RNA as a guide to silence the target messenger RNAs (mRNAs) (Bernstein et al., 2001; Meister & Tuschl, 2004; Tomari & Zamore, 2005; Maiti, Lee, & Liu, 2007; Jinek & Doudna, 2009; Li, Chang, & Liu, 2010). However, the virus can also hijack the host RNAi system to regulate the host’s gene expression (Hu et al., 2013; Miller et al., 2016; Zhao et al., 2016). Plant pathogenic viruses may hijack or induce the host to produce small RNAs for many reasons (Pooggin, 2017). Therefore, by sequencing these small RNAs, it might be possible to identify some specific patterns that could be used as markers for the diagnosis of plant virus disease, and it might even be possible to restore the virus genome from the small RNAs. Detection of viruses using RNA sequence analysis has been performed in a number of different crop plants (Kashif et al., 2012; Li et al., 2012; Wylie et al., 2014; Jo et al., 2015; Jo et al., 2016; Jo et al., 2017; Matsumura et al., 2017).

Many viruses can cause similar disease symptoms in plants. Several different molecular methods have been developed to diagnose plant viral diseases, such as ELISA and RT-PCR, which have increased the speed and accuracy of viral disease diagnosis in crops; however, such techniques only detect expected viruses because each test is specific to one or a small number of related viruses (Mumford et al., 2006; Jones et al., 2017). Mixed infections also cause difficulties when diagnosing virus diseases of plants using traditional diagnostic methods because these methods usually detect one specific virus. There is increasing virus disease happened in *Cucurbita* production of Jinhua. However, because of the disease symptoms are very similar among different virus and viruses infection, the aim of this study was to identify the virus or viruses responsible for the recent disease epidemic of cucurbits in the Jinhua area. We developed a method using all known virus genomes from the NCBI database as a reference to map small RNA without the need for sequence assembly, as is usually required using other virus diagnostic methods. In this study, we identified the newly emerged disease of *Cucurbita* in the Jinhua area of Zhejiang province in China as a mixed infection of three viruses: ZYMV, WMV, and CMV. The disease was diagnosed by sequencing small RNAs from diseased plant samples and further confirmed by RT-PCR and by inoculating healthy plants with infected crude sap extract.

## Materials & Methods

### Survey of the disease

All *Cucurbita* plantations in the Jinhua area were surveyed for disease from 2014 to 2016. The plantation area and the yield loss reported by the producers were recorded. In total, 25 healthy leaves and 25 leaves with symptoms of mosaicking, mottling, blistering, and deformations were collected from healthy and diseased *Cucurbita* plants growing at experimental field sites at the Jihua National Agricultural Technology Garden (29°1′3″N, 119°37′12″E) and stored at −80 °C until ready for analysis.

### Plant material and inoculation

In total, 9 *C. maxima* (cultivar: Miben) plants were planted in a greenhouse with a temperature range of 23 °C to 28 °C, 60% humidity, and natural light. Each single strain of ZYMV, WMV, and CMV was reconstituted from infectious clones as described previously (Feng et al., 2013), and maintained on *Nicotiana tabacum*. Inoculation was carried out by mechanical inoculation (abrade sap mix with SiC particles) on the first true leaves of 14-day-old *C. maxima* seedlings, with sap prepared from leaf tissues of diseased samples from the experimental field of Jihua National Agricultural Technology Garden homogenized in 20 volumes of 100-mM sodium phosphate buffer, pH 7.2. Mock-treated *C. maxima* seedling was inoculated with sodium phosphate buffer only. Single strain sap prepared from *N. tabacum* were used as positive control. Six leaves collected from different *C. maxima* plants showing symptoms of disease were collected.

### Small RNA isolation and sequencing

RNA was extracted from six samples: three leaf samples collected from healthy plants and three leaf samples from diseased plants from the experimental field of Jihua National Agricultural Technology Garden. Total RNA was isolated from each sample using a TRIzol^®^ reagent (Invitrogen, Waltham, MA, USA) and treated with RNase-free DNase I (TaKaRa, Kyoto, Japan) following the manufacturer’s protocol. The integrity of the RNA was verified by running a 1% agarose gel electrophoresis and then staining with ethidium bromide. The concentration of RNA was measured using Nanodrop 2000 (Thermo Fisher Scientific, Waltham, MA, USA).

The purified RNAs were used to construct small RNA libraries according to Illumina’s recommended protocol. Briefly, the purified RNAs were ligated with 3′ and 5′ adapters (Illumina, San Diego, CA, USA). The first strand of complementary DNA (cDNA) was synthesized using reverse transcription and then the synthesized cDNAs were subjected to a PCR amplification process of 15 cycles, with each cycle consisting of the following basic steps: denaturation for 10 s at 98 °C, annealing for 30 s at 60 °C, and extension for 15 s at 72 °C. The amplified products were purified by performing 6% polyacrylamide gel electrophoresis. Following the purification of amplified cDNAs, the products were sequenced using Hiseq2000 (Illumina), and the Illumina SE50 single-end RNA-seq approach, which generated single reads of approximately 50 bp long, which were considered long enough to achieve the full length of the small RNAs. The raw small RNA sequencing data were submitted to the NCBI database under the SRA accession number: PRJNA510225.

### Bioinformatics analysis

Low-quality reads (i.e., reads containing sequencing 5′ adaptors; reads without 3′ adaptors; reads containing 40% nucleotides with a Q quality score lower than 20) were removed. After the removal of low-quality reads, clean reads with 18–40 nt were processed for mapping to virus genome sequences in the NCBI database using bowtie (Langmead et al., 2009; Langmead & Salzberg, 2012). The mapping criteria was set as follows: all quality values were assumed to be 40 on the Phred quality scale; 32 parallel search threads were launched; the maximum number of mismatches permitted in the ”seed” was set as 0; and up to 100 valid alignments per read or pair were reported. All known virus genomes in the NCBI database were indexed and used for mapping. In addition, reads from each sample were assembled using Velvet, using kmer sizes 9, 13, 15, 17, and 23, and using SPAdes, using multi kmer sizes 9, 13, 15, 17, 19, and 23. The results of these assemblies were evaluated based on the size of contigs and N50.

### RT-PCR

RNA samples from the six samples used for small RNA sequencing were also used for RT-PCR validation. In addition, total RNAs were extracted from leaf samples from plants inoculated with infected crude sap and their controls, as previously described for RNA sequencing. The reverse transcription of each RNA sample was performed to obtain cDNA using a PrimeScript RT reagent kit (Invitrogen) with an Oligo dT primer. The RT-PCR was performed in triplicate using a SYBR Premix ExTaq kit (TaKaRa). The specificity of the SYBR green PCR signal was further confirmed by performing a melting curve analysis. Primers used in this study are shown in Table S1. In order to reduce the error, the PCR reaction conditions were optimized by changing the annealing temperature and the annealing time, until their amplification efficiencies were within the range of 95% to 100% (the optimized annealing temperature 56 °C and the annealing time 45 s). After the PCR reaction, the results were visualized by performing gel electrophoresis and staining with ethidium bromide.

## Results

### Disease symptoms and yield loss

**Figure 1:**
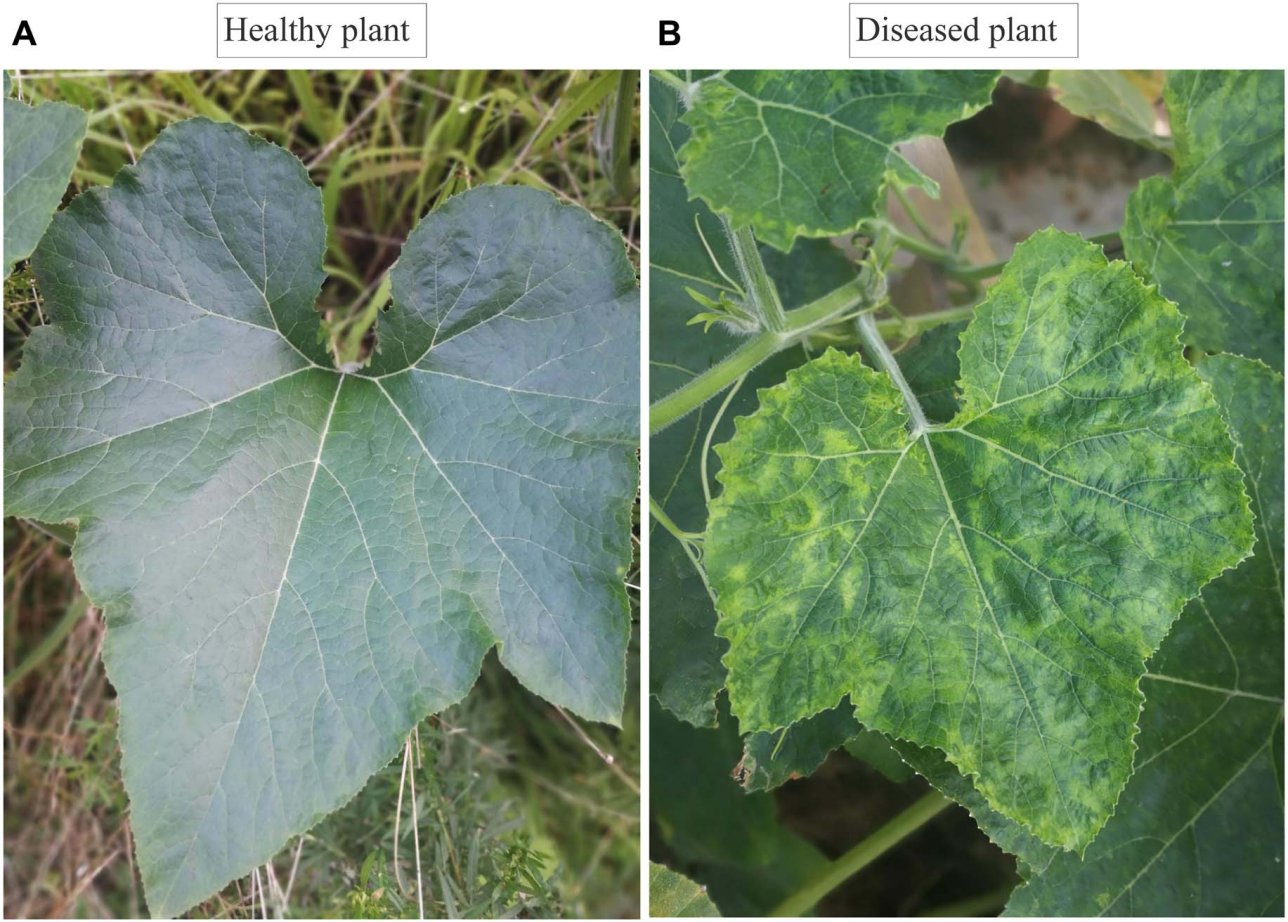
Symptomatic and asymptomatic leaves of *Cucurbita maxima*. (A) Healthy plant leave (B), diseased plant leave from field with mixed infection of *Zucchini yellow mosaic virus*, *Watermelon mosaic virus*, and *Cucumber mosaic virus*.

Viral diseases of *C. maxima* were continually monitored between 2014 and 2016. Leaves of diseased plants generally showed severe leaf mosaicking, yellowing, and eventually ‘shoestring’ symptoms (Fig. 1). In 2014 and 2015, very few plants with disease symptoms were observed in the fields and the disease incidence was approximately 2% and 1%, respectively. In 2016, an outbreak of disease was recorded, with a disease incidence of approximately 50%. The disease caused yield losses of approximately 35%, which were estimated to equate to the loss of 2310 metric tons of *C. maxima* fruit in the Jinhua area (Table 1).

**Table 1:** Disease incidence of *C. maxima* in Jinhua area.

Years	Plantation area in Jinhua (Ha)	Disease incidence (%)	Reduction of yield (%)	Reduction of yield (tons)
2014	366.67	5%		
2015	133.33	8%		
2016	200.00	50%	35%	2310

### Sequencing of small RNA from diseased and healthy plants

Approximately 10 million reads were generated from the three healthy leaf samples and from the three diseased leaf samples. After filtering out low-quality sequences, 98–99% of reads remained and were used for further study (Table 2).

**Table 2:** Reads generated by Small RNA sequencing.

Type	D-1 (Ratio)	D-2 (Ratio)	D-3 (Ratio)	CK-1 (Ratio)	CK-2 (Ratio)	CK-3 (Ratio)
Raw Reads	9532288 (100%)	10652969 (100%)	7835513 (100%)	12812258 (100%)	11431136 (100%)	12647550 (100%)
Clean Reads	9414934 (98.77%)	10570041 (99.22%)	7792080 (99.45%)	12668644 (98.88%)	11272650 (98.61%)	12440926 (98.37%)

**Notes.**

D diseased samples CK healthy samples

### Viral infections detected in *C. maxima* plants

The remaining reads were used to map to all known viral genomes in the NCBI database. Thirty-four different viruses were detected with reads in each diseased samples above 300; however, the majority of reads were mapped to three viruses: ZYMV, WMV, and CMV. On average, approximately 400,000, 200,000 and 229 100 reads were mapped to CMV, ZYMV and WMV, respectively. The remaining reads were mapped to a number of other viruses; however, the number of these mapped reads was very small (Table 3).

**Table 3:** Mapped reads to the viruses in the NCBI database.

Name	D-1	D-2	D-3	CK-1	CK-2	CK-3
Cucumber mosaic virus RNA 1	101269	118734	86556	1	13	21
Cucumber mosaic virus RNA 2	74282	86666	63252	2	7	15
Cucumber mosaic virus RNA 3	205801	239221	173406	5	20	41
Zucchini yellow mosaic virus	190722	223685	164527	8	14	30
Watermelon mosaic virus	120230	138656	101468	12	22	25
Total maped reads/clean reads(%)	8.30%	8.60%	8.51%	0.22%	0.27%	0.24%

**Notes.**

D diseased samples CK healthy samples

Reads from each sample were assembled using Velvet using kmer sizes 9, 13, 15, 17, and 23 and using SPAdes using multi kmer sizes 9, 13, 15, 17, 19, and 23. However, we were unable to assemble the whole genome of each virus. According to the N50 and contig numbers (Table 4), the SPAdes assembly has longest N50 (386 bp), longer total lenth (81,289 bp) and relative less contig number (782). Therefor SPAdes assembly were process for further analysis. In total, four virus were assembled with the coverage above 50% (Table 5). The average coverages of each assembled virus of diseased samples are 99% (Cucumber mosaic virus), 97% (Zucchini yellow mosaic virus), 88% (Watermelon mosaic virus), and 63% Cucurbit aphid-borne yellows virus. The coverage of Cucurbit aphid-borne yellows virus (a member of the Polerovirus genus in the family Luteoviridae) was quite low only around 55–66%, and the virus could not be validated by RT-PCR.

**Table 4:** Reads assembled by different Kmer of Velvet and SPAdes (numbers are average of three diseased samples).

	N50(bp)	Total_length (bp)	Contig Number	Number (50 bp)	Number (2,000 bp)
SPAdes	386	81289	782	334	3
Velvet, Kmer 13	64	31710	2456	474	0
Velvet, Kmer 15	98	100330	1828	1044	0
Velvet, Kmer 17	106	98939	1204	960	0
Velvet, Kmer 19	120	72072	709	629	0
Velvet, Kmer 23	195	16640	109	102	0

**Table 5:** Coverage (%) of assembled virus genomes by SPAdes.

Virus Name	D-1	D-2	D-3	CK-1	CK-2	CK-3
Cucumber_mosaic_virus_RNA_1	99.6	100	98.7	0	0	0
Cucumber_mosaic_virus_RNA_2	99.4	100	97.3	0	0	0
Cucumber_mosaic_virus_RNA_3	96.1	95	98.8	0	0	0
Zucchini_yellow_mosaic_virus	96.7	96.3	98.3	0	0	0
Watermelon_mosaic_virus	87	86.7	91.3	0	0	0
Cucurbit_aphid-borne_yellows_virus	65.9	66.6	55.4	0	0	0

**Notes.**

D diseased samples CK healthy samples

Six *C. maxima* plants mechanically inoculated with crude sap extracted from diseased plants of *C. maxima* (were processed to RT-PCR validation) and three *C. maxima* plants inoculated with crude saps of three single strains of ZYMV, WMV and CMV (were used as positive controls for RT-PCR validation) showed similar symptoms of disease after two weeks (Fig. S1), however the mock-treated *C. maxima* seedling (were used as negative controls for RT-PCR validation) do not show any symptoms.

### RT-PCR validation of a mixed infection of three viruses

**Figure 2:**
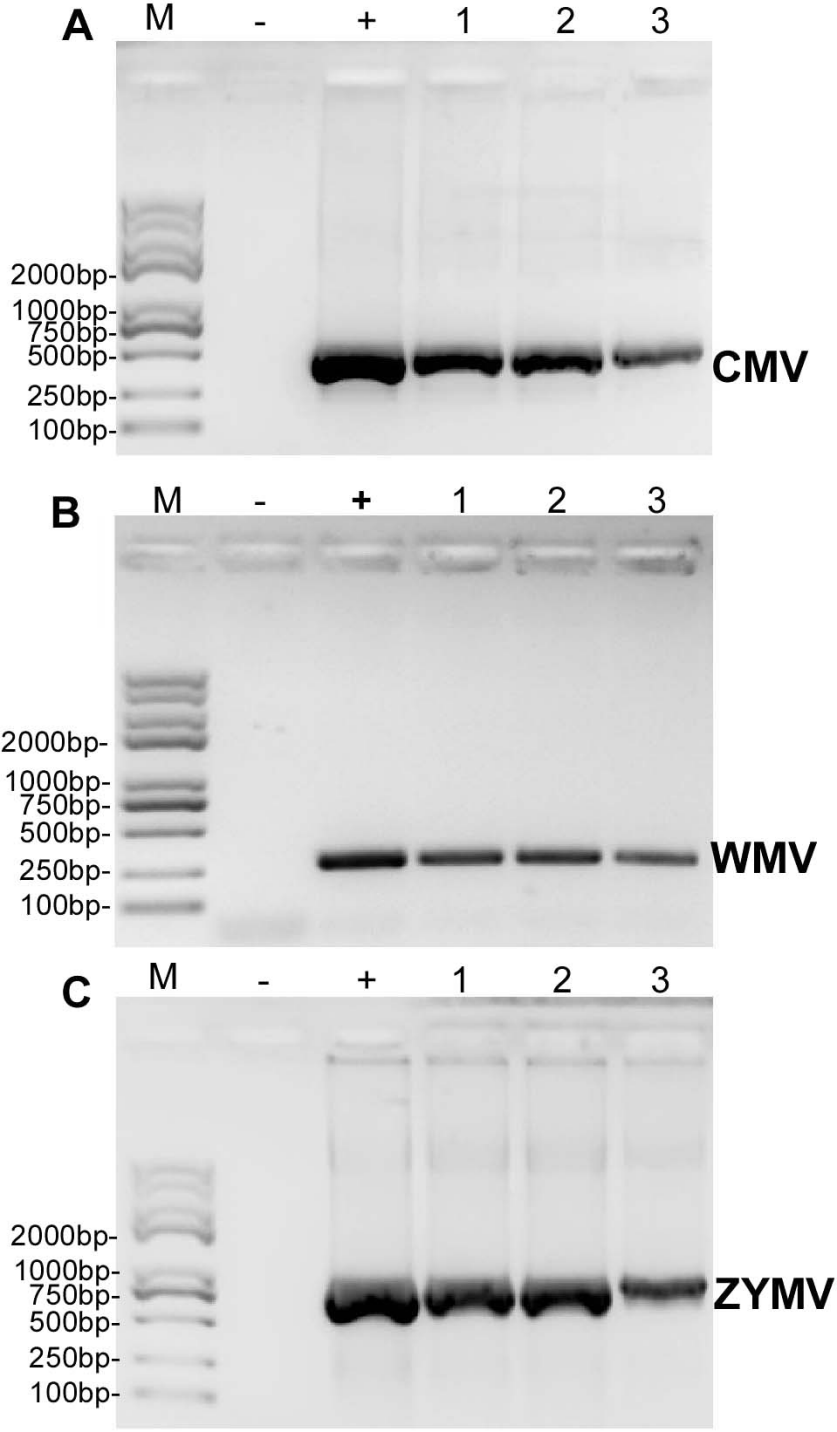
RT-PCR validation of (A) CMV, (B) WMV, and (C) ZYMV in three diseased samples from the experimental field of Jihua National Agricultural Technology Garden. Trans 2K Plus II DNA Marker was used, “+” was positive controls (the plasmid contain virus coat proteins’ sequences preserved in Zhejiang academy of agricultural sciences), “-” was negative controls (healthy plants), “1,2,3” were three replicates of diseased plants from field (which also used for small RNA sequencing).

**Figure 3:**
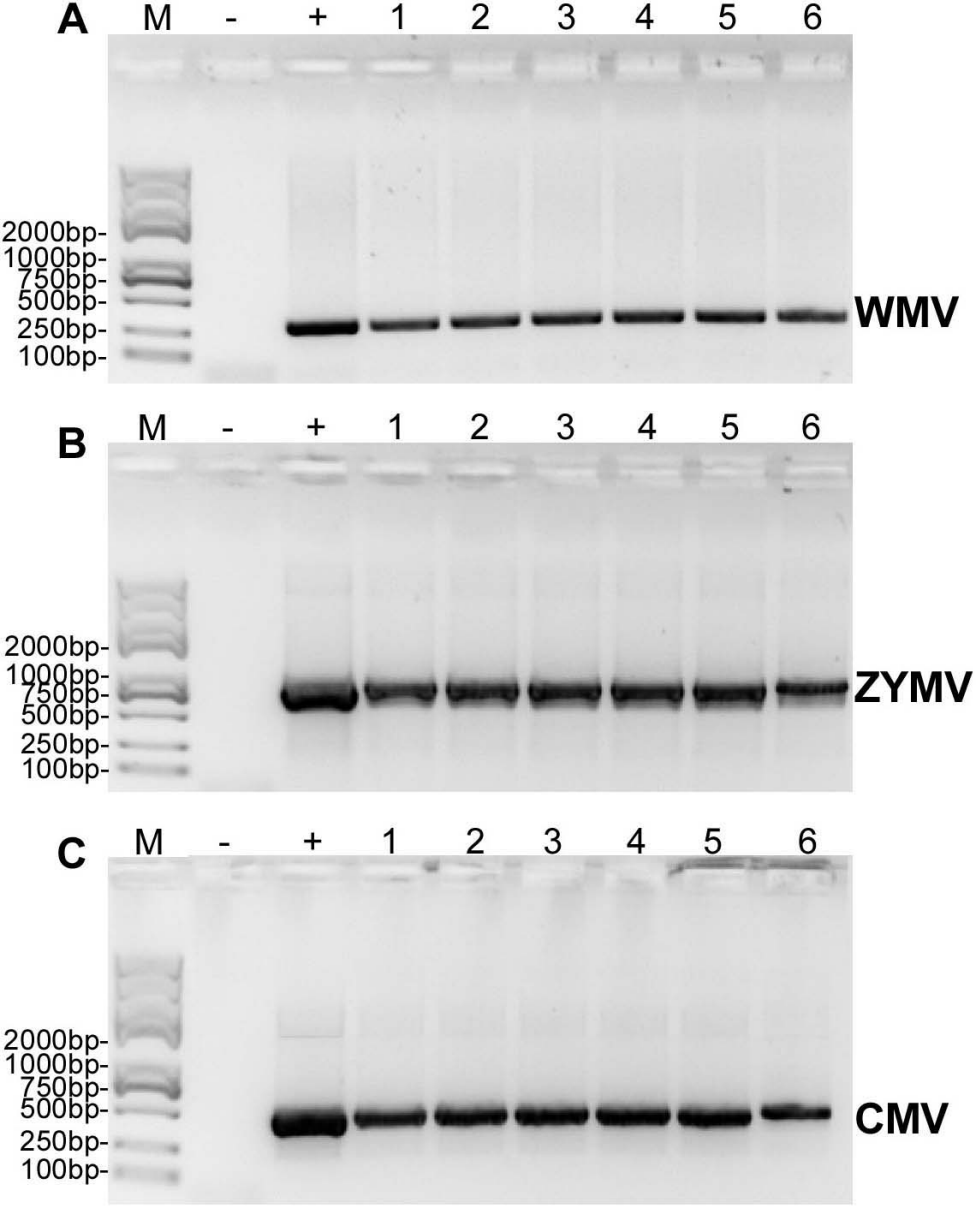
RT-PCR confirmation of the *C. maxima* seedlings in greenhouse mechanically inoculated with crude sap extracted from diseased plants from the experimental field of Jihua National Agricultural Technology Garden. (A) WMV, (B) ZYMV (C) CMV. Trans 2K Plus II DNA Marker was used, “+” was positive controls (single strain sap prepared from *N. tabacum* were used as positive control ), “-” was negative controls (healthy plants), “1,2,3,4,5,6” were six replicates of plants inoculated by sap prepared from leaf tissues of diseased samples from the experimental field of Jihua National Agricultural Technology Garden.

RT-PCR revealed that ZYMV (500 bp amplicon), WMV (247 bp amplicon), and CMV (480 bp amplicon) were present in all diseased samples, but not the asymptomatic samples collected from the experimental field of Jihua National Agricultural Technology Garden (Fig. 2). And in all the leaves of plants inoculated with crude sap extracted from diseased plants, they were detected all three virus as well (Fig. 3).

## Discussion

In this study, we established a method using all known virus genomes from the NCBI database as a reference to map small RNA to diagnose virus diseases of *C. maxima*. In our study, thirty four different viruses were detected with reads above 300 in each diseased samples; however, the majority of the small RNAs were from ZYMV, WMV, and CMV. Mixed infections of these three viruses were diagnosed in leaf samples from diseased plants and further confirmed by real time q-PCR. These three viruses were inoculated together into healthy host plants via infected crude sap extracts. Inoculated plants developed the same disease symptoms as those observed in the diseased plants and a mixed infection of ZYMV, WMV, and CMV was detected again by RT-PCR, thus fulfilling Koch’s postulates. All three viruses were capable of causing leaf mosaicking, yellowing, and eventually “shoestring” symptoms in host leaves.

Mixed viral infections in plants are quite common in nature and results in additive, synergistic or antagonistic interactions that may elicit more severe symptoms than an individual infection by one of the viruses (Mendez-Lozano et al., 2003; Renteria-Canett et al., 2011). However, in this study, it was difficult to determine whether mixed infections induced more severe symptoms than single or co-infections because even single infections caused severe symptoms. Small RNAs from viruses were also detected in healthy plants; however, these were not from plant pathogenic viruses but phages. These results are either likely related to the identification of host genome-integrated viral sequences or a misinterpretation of the results. A drawback of the method used in this study was that although *Cucurbit aphid-borne yellows virus* was found using the assembly method, it could not be validated by RT-PCR. This might be because an error happened during the bioinformatics process, or that the amount of *Cucurbit aphid-borne yellows virus* was too low to be detected by RT-PCR.

Several high-throughput sequencing-based virus detection protocols have previously been developed and evaluated. Massart et al. (2018) compared 21 plant virology laboratories, each employing a different bioinformatics pipeline, to detect 12 plant viruses by testing ten datasets of 21–24 nt small RNA sequences from three different infected plants (Massart et al., 2018). The sensitivity of virus detection ranged between 35% and 100% among participants, the false positive detection rate was very low, and reproducibility was high. However, these studies were all based on bioinformatics strategies for assembling the small reads of the viral genome. In our study, we found that it was difficult to assemble the small RNA reads of the entire virus genome, likely because the small RNA are very short and degraded rapidly in the plant tissue. In addition, assembling the small reads might have been hindered because the amount of sequences we generated was not large enough. However, we showed that sequencing sRNA and mapping the sRNA direct to the viral genomes in the NCBI database was an efficient method of identifying virus species in diseased plants. This method involves fewer bioinformatics processes than other diagnostic methods, does not require complex settings for bioinformatics parameters, provides a high level of sensitivity to rapidly diagnose plant samples with symptoms of virus diseases in the field. Furthermore, the costs associated with this method are reasonable, and should become even cheaper in the future. However, a drawback of this method is that although sequences for new (non-reported viruses) were found in this study, they could not be identified because their genome information was not present in the NCBI database, therefore, sequence assembly and further molecular validation are still required for new viruses.

## Conclusions

The diagnostic method used in this study was a very effective way of diagnosing the mixed virus infection in diseased *Cucurbita* plants and has potential to be widely applied as a diagnostic method for viruses that have genome information in the NCBI database.

##  Supplemental Information

10.7717/peerj.7930/supp-1 Figure S1 The disease symptoms with infection of virus ZYMV alone, virus WMV alone, and virus CMV alone

10.7717/peerj.7930/supp-2 Table S1 List of specific real-time QPCR primers for three identified viruses

10.7717/peerj.7930/supp-3 Supplemental Information 2 The assembled viruses sequences from diseased samples

## Additional Information and Declarations

### Data Availability

The following information was supplied regarding data availability: Data is available in the NCBI database: PRJNA510225
