# Supplementary figures and images for: Detection of disease in Cucurbita maxima Duch. ex Lam. caused by a mixed infection of Zucchini yellow mosaic virus, Watermelon mosaic virus, and Cucumber mosaic virus in Southeast China using a novel small RNA sequencing method
Source: PeerJ. 2019 Oct 23;7:e7930. doi: 10.7717/peerj.7930 (PMC6815192; doi:10.7717/peerj.7930)

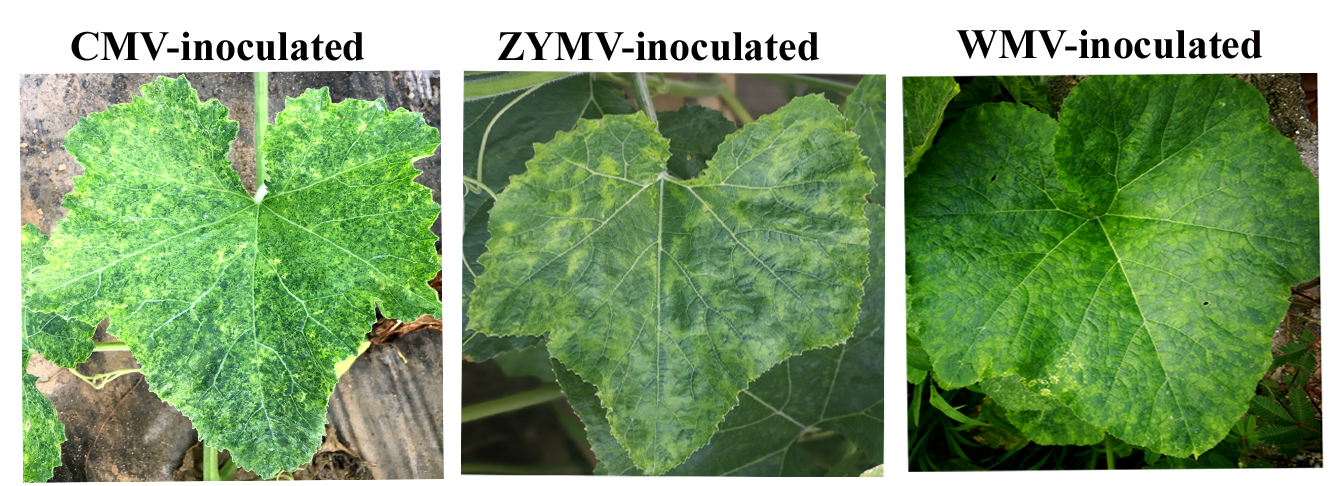

Supplement: Figure S1 [file peerj-07-7930-s001.jpg]
