# Supplementary material for: Detection of disease in Cucurbita maxima Duch. ex Lam. caused by a mixed infection of Zucchini yellow mosaic virus, Watermelon mosaic virus, and Cucumber mosaic virus in Southeast China using a novel small RNA sequencing method
Source: PeerJ. 2019 Oct 23;7:e7930. doi: 10.7717/peerj.7930 (PMC6815192; doi:10.7717/peerj.7930)
Supplement: Table S1 [file peerj-07-7930-s002.docx]

Table S1. List of specific real-time QPCR primers for three identified viruses

| Primer Names | Primer Sequences | Size of products |
| --- | --- | --- |
| WMV-N | 5’- ATGCAGGAAAGGATTCGAAGA-3’ | 247bp |
| WMV-C | 5’-TCAACTTGATTAGGCTTGTAT-3’ |  |
| ZYMV-N: | 5’-ATGGTTGTCATTTCGATATAT-3’ | 500bp |
| ZYMV-C: | 5’-TTATCAGTATACAACTTCCGG-3’ |  |
| CMV-N | 5’-ATGGATAAATCTGAATCAACC-3’ | 480bp |
| CMV-C | 5’-TTGGACTCCAGATGCGGCATA-3’ |  |
